# Cohort study on the prognosis of acute cerebral infarction in different circulatory systems at 1-year follow-up

**DOI:** 10.1186/s12872-021-02291-0

**Published:** 2021-10-29

**Authors:** Li-Li Chen, Wen-Ting Wang, Sai Zhang, Hui-Miao Liu, Xiao-Yang Yuan, Xu Yang, Ping Gu

**Affiliations:** 1 Department of Neurology, The First Hospital of Hebei Medical University, Shijiazhuang, 050031 Hebei China; 2 Department of Neurology, Aerospace Center Hospital, Peking University Aerospace School of Clinical Medicine, Beijing, 100049 China

**Keywords:** Acute cerebral infarction, Anterior circulation, Posterior circulation, Prognosis, Prognostic factor

## Abstract

**Background:**

To evaluate the prognosis of acute cerebral infarction at 1-year follow-up in different circulation infarctions.

**Methods:**

Clinical data of 858 consecutive patients with acute cerebral infarction were collected. Of the 858 cases, 21 (2.45%) were lost to follow-up and 837 completed follow-up and thus were enrolled in this study. At 1-year follow-up, death or moderate-to-severe dysfunction (modified Rankin Scale (mRS) ≥ 3 points) was regarded as the poor prognostic endpoint. Univariate analysis and multivariate logistic stepwise regression analysis were performed to assess the prognosis. The prediction probability of indicators was obtained for the multivariate model, and the receiver operating characteristic curve was delineated to calculate the area under the curve (AUC) to predict the fitness of the model.

**Results:**

The older the age, the greater the probability of a poor prognosis. Patients with previous diabetes and cerebral infarction had a poor prognosis. The higher the National Institutes of Health Stroke Scale and mRS scores and the lower the Barthel index at admission, the worse the prognosis of the patients. The longer the hospital stay, the worse the prognosis of the patients. The prognosis of different circulation infarctions was different. The AUC of the multivariate model was AUC = 0.893, and the 95% confidence interval was 0.870–0.913, indicating a good fit. The prognosis of anterior circulation infarction (ACI) was worse than that of posterior circulation infarction (PCI) (*P* < 0.05). The prognosis of patients with ACI and PCI was not significantly different from that of patients with ACI or PCI alone (*P* > 0.05).

**Conclusions:**

Diabetes, the Barthel index at admission and previous cerebral infarction are poor prognostic factors of acute cerebral infarction. The prognosis of ACI is worse than that of PCI. Different factors affect the prognosis of different circulatory system infarctions.

## Background

Acute cerebral infarction accounts for approximately 85% of all strokes and has a high disability and mortality rate [1, 2]. Therefore, the prevention and treatment of acute cerebral infarction are important in this respect. Few clinical studies compared the aetiology and prognostic factors of different circulation strokes, and variable analyses are unavailable [3, 4]. Initial stroke severity, age, consciousness level, hyperglycaemia and stroke mechanisms are related to the prognosis after acute cerebral infarction, which may differ between anterior circulation infarction (ACI) and posterior circulation infarction (PCI) [5, 6]. The present study aimed to evaluate the prognostic factors of acute cerebral infarction at 1-year follow-up in different circulation infarctions.

## Methods

### Study object

This prospective cohort study was approved by local ethics associations and hospital ethics committees. All methods were performed in accordance with the ethical guidelines and regulations. This study was carried out in compliance with the STROBE guidelines. Data were consecutively collected on 858 patients with acute cerebral infarction who were hospitalised in the Department of Neurology, Aerospace Center Hospital, Peking University Aerospace School of Clinical Medicine from December 1, 2012 to November 4, 2015. Data included demographics, medial history, self-care ability before admission, stroke severity, biochemical indicators and imaging manifestations.

Inclusion criteria:

1. Age > 18 years;
2. Acute cerebral infarction which was initially diagnosed in accordance with World Health Organization standards and confirmed by brain MRI;
3. Time from onset to hospital admission ≤ 14 days;

The aetiology of each patient was grouped in accordance with TOAST classification (large atherosclerosis, small artery occlusion, cardiogenic embolism and other types with clear or unknown causes). Written informed consent was obtained from all patients enrolled or their next of kin.

Exclusion criteria:

1. Patients with haemorrhagic stroke or TIA;
2. Asymptomatic cerebral infarction;
3. Non-cerebrovascular disease events, such as primary brain tumours, subdural haemorrhage, Todd’s palsy, etc.;
4. Time interval from onset to hospital admission > 14 days;
5. Those who had not signed the informed consent.

### Data collection

In this prospective cohort study, baseline data were collected by professionally trained neurologists through face-to-face interviews, and those data in the paper-based case report form were entered by trained personnel. The medical records of patients meeting the inclusion criteria were used to establish an electronic database with Epidata3.1 software, record data and information, and implement parallel double entry, including (1) basic information: gender, age, marital status, education level, occupation; (2) past medical and personal history: hypertension, diabetes, heart disease, cerebrovascular disease history (cerebral infarction, cerebral haemorrhage, transient ischaemic attack and so on), history of cardiogenic diseases (chronic heart failure, atrial fibrillation, coronary heart disease and so on), cancer, history of smoking, history of drinking and so on; (3) clinical features: time from onset to hospital admission, baseline US National Institutes of Health Stroke Scale (NIHSS) score, modified Rankin Scale (mRS) score, Barthel index and so on; (4) laboratory data: haemoglobin, blood glucose, blood lipids, renal function and so on; (5) imaging data and TOAST classification; (6) clinical data at 3-, 6-, 9- and 12-month follow-up.

### Follow-up and outcome events

Follow-up visits were conducted by doctors over the phone or through face-to-face interviews. Telephone follow-up was performed by well-trained interviewers. Since the onset, the patients were followed up for 3, 6, 9 and 12 months with a time deviation of no more than 7 days. The primary outcome was poor prognosis, defined as ① death (after discharge) with acute cerebral infarction as the direct or indirect cause of death and ② moderate-to-severe disability and inability of self-care, that is, mRS ≥ 3 points.

### Statistical methods

A normal distribution test was initially performed to compare the baseline characteristics which did not conform to a normal distribution. Continuous variables were represented by median and interquartile ranges, and non-parametric dual independent sample tests were conducted. For categorical variables, a χ^2^ test was applied. Yates continuity correction or Fisher’s exact probability test was carried out if necessary. Single-factor analysis was performed on the previously described data. Multivariate logistic stepwise regression analysis was performed with the index of *P* < 0.1. Prediction probabilities of the indexes were obtained for the multivariate model. Medcalc software was used to delineate the receiver operating characteristic curve (ROC) and calculate the area under the curve (AUC) to predict the fitness of the model. On the basis of the different circulatory systems of cerebral infarction, a multivariate logistic stepwise regression analysis was performed to evaluate the prognostic factors. Statistical analysis was performed using SPSS 25.0 (SPSS Inc., Chicago, IL) and MedCalc software with a two-tailed test, and statistical significance was considered at *P* < 0.05.

## Results

### General

A total of 858 patients met the inclusion criteria, of whom 21 cases (2.45%) were lost to follow-up and 837 completed follow-up and thus were enrolled in this study. Some data were missing in the profile, including haemoglobin in 19 patients, triglycerides in 19 patients, low-density lipoprotein in 19 patients, high-density lipoprotein in 19 patients, cholesterol in 19 patients, blood glucose in 17 patients, creatinine in 17 patients and urea nitrogen in 17 patients. Smoking and drinking were classified data. Each was divided into several levels. For example, smoking was divided into four levels. The number of patients in each level added up to the total number of patients in each group. One level comprised 437 non-smokers: 316 (72.3%) in the good prognosis group and 121 (27.7%) in the poor prognosis group.

### **Univariate and multivariate analysis**

Univariate analysis revealed poor prognostic factors (*P* < 0.1), as shown in Table 1.

**Table 1:** Univariate analysis of baseline characteristics and laboratory indicators of patients with acute cerebral infarction

Clinical features and laboratory indicators	Good prognosis (n = 630)	Poor prognosis (n = 207)	Non-parametric test(Z or X2 )value	*P* value
Age (years), M(QR)	64 (21)	74 (16)	− 8.130	0.000
Admission NIHSS, M(QR)	2 (4)	8 (9)	− 13.315	0.000
Barthel index, M(QR)	90 (30)	35 (50)	− 14.781	0.000
Hemoglobin (g/L), M(QR)	141 (22)	136 (27)	− 3.972	0.000
Triglycerides (mmol/L), M(QR)	1.40 (1.04)	1.19 (1.09)	− 2.417	0.016
Cholesterol (mmol/L), M(QR)	4.56 (1.34)	4.34 (1.83)	− 1.652	0.098
Low-density lipoprotein (mmol/L), M(QR)	2.59 (1.04)	2.45 (1.42)	− 1.933	0.053
High density lipoprotein (mmol/L), M(QR)	0.75 (0.24)	0.75 (0.28)	− 0.311	0.756
Blood sugar (mmol/L), M(QR)	6.58 (3.46)	7.30 (4.28)	− 3.150	0.002
Creatinine (umol/L), M(QR)	71.10 (23.28)	73.20 (27.80)	− 2.182	0.029
Urea nitrogen (umol/L), M(QR)	5.30 (2.23)	6.00 (3.10)	− 4.174	0.000
Gender, n(%)			5.034	0.025
Female	182 (28.9%)	77 (37.2%)		
Male	448 (71.1%)	130 (62.8%)		
History				
Diabetes, n(%)			7.394	0.007
No	436 (69.2%)	122 (58.9%)		
Yes	194 (30.8%)	85 (41.1%)		
Hypertension, n(%)			3.615	0.057
No	225 (35.7%)	59 (28.5%)		
Yes	405 (64.3%)	148 (71.5%)		
Hyperlipidemia, n(%)			0.260	0.610
No	527 (83.7%)	170 (82.1%)		
Yes	103 (16.3%)	37 (17.9%)		
Smoking, n(%)			10.294	0.016
No smoking	316 (50.2%)	121 (58.5%)		
Quit smoking	67 (10.6%)	26 (12.5%)		
Still smoking	240 (38.1%)	55 (26.6%)		
Unknown	71 (1.1%)	5 (2.4%)		
Drinking, n(%)			15.959	0.014
No drinking	407 (64.6%)	159 (76.8%)		
Drink moderately	146 (23.2%)	28 (13.5%)		
Moderate drinking	33(5.2%)	6 (2.9%)		
Heavy drinking	10(1.6%)	1 (0.5%)		
Unknown alcohol consumption	12(1.9%)	2 (1.0%)		
Quit alcohol	19 (3.0%)	9 (4.3%)		
Unknown	3 (0.5%)	2 (1.0%)		
Cerebral Infarction, n(%)			39.796	0.000
No	480 (76.2%)	110 (53.1%)		
Yes	150 (23.8)	97 (46.9%)		
Cerebral hemorrhage, n(%)			9.937	0.002
No	616 (97.8%)	193 (93.2%)		
Yes	14 (2.2%)	14 (6.8%)		
TIA, n(%)			3.256	0.071
No	623 (98.9%)	201 (97.1%)		
Yes	7 (1.1%)	6 (2.9%)		
Heart failure, n(%)			4.176	0.041
No	624 (99.0%)	201 (97.1%)		
Yes	6 (1.0%)	6 (2.9%)		
Coronary Heart Disease, n(%)			6.182	0.013
No	529 (84.0%)	158 (76.3%)		
Yes	101 (16.0%)	49 (23.7%)		
Atrial fibrillation, n(%)			12.796	0.000
No	589 (93.5%)	177 (85.5%)		
Yes	41 (6.5%)	30 (14.5%)		
Tumor, n(%)			10.673	0.001
No	615 (97.6%)	192 (92.8%)		
Yes	15 (2.4%)	15 (7.2%)		
MRS at admission, n(%)			263.515	0.000
0	13 (2.1%)	2 (1.0%)		
1	175 (27.8%)	6 (2.9%)		
2	192 (30.5%)	24 (11.6%)		
3	154 (24.4%)	36 (17.4%)		
4	87 (13.8%)	77 (37.2%)		
5	9 (1.4%)	62 (30.0%)		
Length of hospital stay, n(%)			124.628	0.000
< 2 weeks	402 (63.8%)	67 (32.4%)		
≥ 2 weeks and < 4 weeks	214 (34.0%)	92 (44.4%)		
≥ 4 weeks	14 (2.2%)	48 (23.2%)		
TOAST type, n(%)			48.677	0.000
Atherosclerotic stroke	333 (52.9%)	104 (50.2%)		
Cardiogenic embolism	20 (3.2%)	27 (13.0%)		
Arteriole occlusive stroke	133 (21.1%)	14 (6.8%)		
Uncertain cause	144 (22.8%)	62 (30.0%)		
Circulatory system, n(%)			21.415	0.000
Anterior circulatory system	387 (61.4%)	151 (72.9%)		
Posterior circulatory system	207 (32.9%)	35 (16.9%)		
Anterior and posterior circulatory system	36 (5.7%)	21 (10.2%)		

Continuous variables are represented by the median (interquartile range), and categorical variables are represented by the number of cases (percentage of the two groups)

Further multivariate logistic regression analysis was performed on the above poor prognosis (P < 0.1), and adjusting factors such as gender, smoking, drinking and diabetes were included. The results are shown in Table 2. The older the age, the greater the probability of a poor prognosis. Patients with previous diabetes and cerebral infarction had a poor prognosis. The higher the NIHSS and mRS scores at and the lower the Barthel index at admission, the worse the prognosis of patients; the longer the hospital stay, the worse the prognosis of the patients. The prognosis of different circulation infarctions was different. ACI has worse prognosis than PCI (*P* < 0.05); the prognosis of the patients with ACI–PCI did not differ significantly from that of the patients with ACI or PCI alone (*P* > 0.05).

**Table 2:** Multivariate logistic analysis for prognostic factors of acute cerebral infarction at 1-year follow-up

Prognostic factors	B	Standard error	Wald	Significance	Exp(B)	95% CI of EXP(B)	
Age	0.049	0.010	24.863	0.000	1.050	1.030	1.070
History of diabetes	0.570	0.228	6.227	0.013	1.768	1.130	2.767
Infarction	0.981	0.227	18.674	0.000	2.666	1.709	4.160
Admission MRS	0.324	0.161	4.053	0.044	1.382	1.009	1.894
Admission NIHSS	0.068	0.032	4.602	0.032	1.071	1.006	1.139
Barthel index	− 0.024	0.007	11.003	0.001	0.976	0.963	0.990
Circulatory system^a^			8.461	0.013			
1	0.779	0.269	8.373	0.004	2.180	1.286	3.695
3	0.358	0.489	0.534	0.465	1.430	0.548	3.732
Circulatory system^b^			8.461	0.013			
2	− 0.779	0.269	8.373	0.004	0.459	0.271	0.778
3	− 0.422	0.450	0.877	0.349	0.656	0.272	1.585
Hospital stay	0.794	0.176	20.475	0.000	2.213	1.569	3.121

Circulatory system^a^ is the control of posterior circulatory infarction

Circulatory system^b^ is the control of the anterior circulation infarction

1 = Anterior circulatory system

2 = Posterior circulatory system

3 = Anterior and posterior circulatory system

### ROC curve

**Fig. 1:**
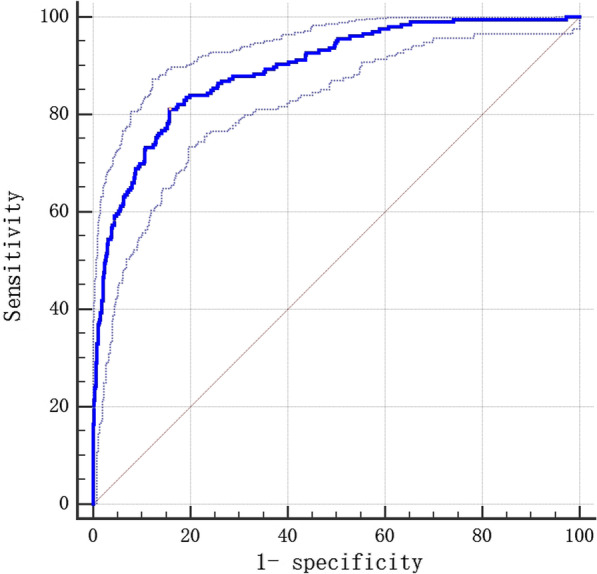
ROC curve of logistic multivariate prediction model for the prognosis of acute cerebral infarction patients at 1-year follow-up AUC = 0.893, *P* < 0.001, Youden IndexJ = 0.6535, Specificity = 84.29, Sensitivity = 81.07

The predicted probabilities of poor prognosis indicators for the multivariate model were calculated. MedCalc software was used to delineate ROC and calculate the AUC to predict the fitness of the model (AUC = 0.893, 95% confidence interval [CI], 0.870–0.913), as shown in Fig. 1.

### Different circulation infarctions

The number of patients with ACI was 538, including 521 with infarction in the middle cerebral artery and 17 in the anterior cerebral artery. Meanwhile, the number of patients with PCI was 242, including 105 with infarction in the posterior cerebral artery, 46 in the basilar artery, 16 in the pontine branch, 17 in the superior cerebellar artery, 5 in the anterior inferior artery and 53 in the posterior inferior cerebellar artery. The number of patients with ACI-PCI was 57.

### Group analysis

Univariate analysis according to different circulatory systems of cerebral infarctions had different prognostic factors. The results are shown in Table 3 (*P* < 0.1).

**Table 3:** Univariate analysis of baseline characteristics and laboratory indicators in patients with ACI andPCI

Clinical features and laboratory indicators	Good prognosis in ACI (n = 387)	Poor prognosis in ACI (n = 151)	Non-parametric test (Z or X2 value)	*P* value	Good prognosis in PCI (n = 207)	Poor prognosis in PCI (n = 35)	Non-parametric test (Z or X2 value)	*P* valve
Age (years), M(QR)	64 (22)	74 (16)	− 6.964	0.000	63 (19)	74 (15)	− 3.286	0.001
Admission NIHSS, M(QR)	3 (4)	8 (8)	− 10.742	0.000	2 (3)	6 (10)	− 5.291	0.000
Barthel index, M(QR)	90 (30)	40 (50)	− 12.124	0.000	90 (30)	50 (60)	− 5.991	0.000
Hemoglobin (g/L), M(QR)	141 (22)	136 (25)	− 2.743	0.006	141 (18)	138 (31)	− 1.466	0.148
Triglycerides (mmol/L), M(QR)	1.395 (1.700)	1.190 (1.750)	− 2.260	0.024	1.390 (1.030)	1.510 (1.250)	− 0.255	0.799
Cholesterol (mmol/L), M(QR)	4.490 (1.350)	4.540 (1.885)	− 0.262	0.793	4.640 (1.410)	4.270 (1.840)	− 1.078	0.281
Low-density lipoprotein (mmol/L), M(QR)	2.580 (1.098)	2.520 (1.430)	− 0.805	0.421	2.570 (1.050)	2.480 (1.450)	− 0.877	0.380
High density lipoprotein (mmol/L), M(QR)	0.745 (0.230)	0.760 (0.305)	− 1.132	0.258	0.750 (0.270)	0.720 (0.260)	− 0.533	0.594
Blood sugar (mmol/L), M(QR)	6.470 (3.205)	7.025 (4.218)	− 2.703	0.007	6.945 (3.980)	8.120 (4.500)	− 1.842	0.065
Creatinine (umol/L), M(QR)	71.000 (24.000)	72.800 (29.700)	− 1.507	0.132	71.100 (21.600)	77.700 (29.600)	− 2.231	0.026
Urea nitrogen (umol/L), M(QR)	5.300 (2.350)	6.000 (3.125)	− 3.427	0.001	5.400 (2.075)	6.000 (3.800)	− 2.187	0.029
Gender, n(%)			4.675	0.031			0.002	0.960
Female	109 (28.2%)	57 (37.7%)			60 (29.0%)	10 (28.6%)		
Male	278 (71.8%)	94 (62.3%)			147 (71.0%)	25 (71.4%)		
History								
Diabetes, n(%)			4.412	0.036			7.710	0.005
No	277 (71.6%)	94 (62.3%)			134 (64.7%)	14 (40.0%)		
Yes	110 (28.4%)	57 (37.7%)			73 (35.3%)	21 (60.0%)		
Hypertension, n(%)			1.069	0.301			3.669	0.055
No	144 (37.2%)	49 (32.5%)			69 (33.3%)	6 (17.1%)		
Yes	243 (62.8%)	102 (67.5%)			138 (66.7%)	29 (82.9%)		
Hyperlipidemia, n(%)			0.069	0.792			0.601	0.438
No	324 (83.7%)	125 (82.8%)			171 (82.6%)	27 (77.1%)		
Yes	63 (16.3%)	26 (17.2%)			36 (17.4%)	8 (22.9%)		
Smoking, n(%)			9.009	0.029			0.981	0.806
No smoking	187 (48.3%)	86 (57.0%)			108 (52.2%)	19 (54.3%)		
Quit smoking	36 (9.3%)	18 (11.9%)			28 (13.5%)	6 (17.1%)		
Still smoking	160 (41.4%)	43 (28.5%)			68 (32.9%)	10 (28.6%)		
Unknown	4 (1.0%)	4 (2.6%)			3 (1.4%)	0 (0.0%)		
Drinking, n(%)			11.319	0.079			4.865	0.561
No drinking	252 (65.1%)	117 (77.4%)			131 (63.3%)	25 (71.4%)		
Drink moderately	90 (23.2%)	20 (13.2%)			47 (22.7%)	7 (20.0%)		
Moderate drinking	23 (5.9%)	6 (4.0%)			9 (4.4%)	0 (0.0%)		
Heavy drinking	6 (1.6%)	1 (0.7%)			4 (1.9%)	0 (0.0%)		
Unknown alcohol consumption	7 (1.8%)	1 (0.7%)			5 (2.4%)	0 (0.0%)		
Quit alcohol	8 (2.1%)	5 (3.3%)			9 (4.3%)	3 (8.6%)		
Unknown	1 (0.3%)	1 (0.7%)			2 (1.0%)	0 (0.0%)		
Cerebral Infarction, n(%)			18.812	0.000			8.944	0.003
No	284 (73.4%)	82 (54.3%)			166 (80.2%)	20 (57.1%)		
Yes	103 (26.6%)	69 (45.7%)			41 (19.8%)	15 (42.9%)		
Cerebral hemorrhage, n(%)			9.636	0.002			0.127	0.722
No	377 (97.4%)	138 (91.4%)			203 (98.1%)	34 (97.1%)		
Yes	10% (2.6%)	13 (8.6%)			4 (1.9%)	1 (2.9%)		
TIA, n(%)			0.243	0.622				0.269
No	381 (98.4%)	147 (97.4%)			206 (99.5%)	34 (97.1%)		
Yes	6 (1.6%)	4 (2.6%)			1 (0.5%)	1 (2.9%)		
Heart failure, n(%)			1.690	0.194				0.467
No	384 (99.2%)	147 (97.4%)			204 (98.6%)	34 (97.1%)		
Yes	3 (0.8%)	4 (2.6%)			3 (1.4%)	1 (2.9%)		
Coronary Heart Disease, n(%)			3.894	0.048			0.011	0.915
No	321 (82.9%)	114 (75.5%)			176 (85.0%)	30 (85.7%)		
Yes	66 (17.1%)	37 (24.5%)			31 (15.0%)	5 (14.3%)		
Atrial fibrillation, n(%)			4.366	0.037			0.252	0.615
No	360 (93.0%)	132 (87.4%)			197 (95.2%)	32 (91.4%)		
Yes	27 (7.0%)	19 (12.6%)			10 (4.8%)	3 (8.6%)		
Tumor, n(%)			10.072	0.002			0.312	0.576
No	380 (98.2%)	140 (92.7%)			200 (96.6%)	35 (100.0%)		
Yes	7 (1.8%)	11 (7.3%)			7 (3.4%)	0 (0.0%)		
MRS at admission, n(%)			159.495	0.000			60.548	0.000
0	10 (2.6%)	2 (1.3%)			2 (1.0%)	0 (0.0%)		
1	94 (24.3%)	5 (3.3%)			76 (36.7%)	1 (2.9%)		
2	128 (33.1%)	18 (11.9%)			55 (26.6%)	5 (14.3%)		
3	95 (24.5%)	27 (17.9%)			45 (21.7%)	7 (20.0%)		
4	54 (14.0%)	58 (38.4%)			27 (13.0%)	14 (40.0%)		
5	6 (1.5%)	41 (27.2%)			2 (1.0%)	8 (22.8%)		
Length of hospital stay, n(%)			78.143	0.000			30.651	0.000
< 2 weeks	257 (66.4%)	51 (33.8%)			125 (60.4%)	12 (34.3%)		
≥ 2 weeks and < 4 weeks	123 (31.8%)	70 (46.3%)			75 (36.2%)	13 (37.1%)		
≥ 4 weeks	7 (1.8%)	30 (19.9%)			7 (3.4%)	10 (28.6%)		
TOAST type, n(%)			33.715	0.000			8.654	0.034
Atherosclerotic stroke	204 (52.7%)	75 (49.7%)			106 (51.2%)	20 (57.1%)		
Cardiogenic embolism	12 (3.1%)	13 (8.6%)			5 (2.4%)	4 (11.4%)		
Arteriole occlusive stroke	95 (24.6%)	11 (7.3%)			37 (17.9%)	3 (8.6%)		
Uncertain cause	76 (19.6%)	52 (34.4%)			59 (28.5%)	8 (22.9%)		

Continuous variables are represented by the median (interquartile range), and categorical variables are represented by the number of cases (percentage of the two groups)

Covariates of the multivariate analysis of the prognosis of different circulation infarctions were entered in accordance with the abovementioned undivided system to balance the differences in matching. Different circulatory systems had different poor prognosis factors. The results are shown in Tables 4, 5 and 6.

**Table 4:** Multivariate logistic analysis for prognosis of patients with ACI at 1-year follow-up

Prognostic factors	B	Standard error	Wald	Significance	Exp(B)	95% CI of EXP(B)	
						Lower limit	Upper limit
Age	0.050	0.011	20.282	0.000	1.052	1.029	1.075
Cerebral infarction history	1.019	0.265	14.774	0.000	2.771	1.648	4.659
NIHSS at admission	0.109	0.051	4.640	0.031	1.115	1.010	1.232
Length of hospital stay	0.793	0.213	13.884	0.000	2.211	1.456	3.356
Barthel index	− 0.030	0.007	16.628	0.000	0.970	0.956	0.984

**Table 5:** Multivariate logistic analysis for prognosis of patients with PCI at 1-year follow-up

Prognostic factors	B	Standard error	Wald	Significance	Exp(B)	95% CI of EXP(B)	
						Lower limit	Lower limit
Cerebral infarction history	0.976	0.494	3.900	0.048	2.655	1.007	6.995
MRS at admission	0.709	0.319	4.946	0.026	2.032	1.088	3.795
Length of hospital stay	0.785	0.349	5.056	0.025	2.193	1.106	4.349

**Table 6:** Multivariate logistic analysis for prognosis with ACI-PCI at 1-year follow-up

Prognostic factors	B	Standard error	Wald	Significance	Exp(B)	95% CI of EXP(B)	
						Lower limit	Lower limit
Cerebral infarction history	2.097	0.982	4.561	0.033	8.144	1.188	55.820
Hemoglobin	− 0.039	0.019	3.915	0.048	0.962	0.926	1.000
Barthel index	− 0.058	0.017	11.908	0.001	0.943	0.913	0.975

## Discussion

Acute cerebral infarction has high disability and mortality. Early prediction of long-term adverse outcomes of patients with acute cerebral infarction based on prognostic factors can help guide early intervention to obtain the best possible outcome and quality of life for patients with acute cerebral infarction. This prospective cohort study analysed the poor rains of patients with acute cerebral infarction during the 1-year follow-up and compared the prognoses of patients with different circulatory infarctions.

Age, NIHSS and mRS scores are closely related to the prognosis of cerebral infarction. The structure and function of blood vessels change with age, which could damage the function of vascular endothelial cells. The latter plays a vital role in the occurrence and development of stroke [7]. The higher the scores of NIHSS and mRS, the more severe the neurological damage and the worse the prognosis. Shortening the length of hospital stay can reduce the occurrence of complications and the psychological stress and depression of patients, thereby improving the prognosis. Early rehabilitation is a cost-effective and safe program which allows severely ill survivors to be discharged early and improve functional recovery [8]. Therefore, the longer the hospital stay, the worse the prognosis of patients. The results of this study are the same.

Diabetes is an independent risk factor for acute cerebral infarction. Patients with diabetes are prone to vascular injury, and the decrease in vascular elasticity affects their contraction function, thereby reducing the perfusion level of the ischaemic penumbra [9]. Long-term hyperglycaemia can also damage vascular endothelial cells, leading to increased circulatory dysfunction and ischaemia [10]. The results of this study showed that diabetes is a poor prognostic factor for acute ischemic stroke. The Barthel index score as a scoring system has a high predictive value for the prognosis of patients with acute cerebral infarction; the lower the score, the worse the prognosis [11]. The results of this study are consistent with them. Our study found that the patient’s medical history of cerebral infarction is a predictive indicator of a poor prognosis of acute cerebral infarction. In addition, the poor prognosis of different circulatory system infarctions plays a role. Studies have shown that patients with asymptomatic carotid artery stenosis and cerebral infarction have a higher risk of stroke recurrence and a poor prognosis during 10-year long-term follow-up [12].

At present, few studies compared the long-term adverse prognoses of patients with acute ACI or PCI. According to reports, the mortality rate of PCI is higher than 25%, and the risk of PCI recurrence is higher than that of anterior circulation ischaemia [13]. However, the development of neuroimaging has promoted the research of PCI diagnosis, and great progress has been achieved in the treatment of cerebrovascular diseases, thereby improving the prognosis of PCI [14, 15]. The NEMC-PCR Center’s research shows that 78.7% of patients with PCI have a good prognosis [16]. Another study showed that the 1-month, 3-month and 1-year mortality rates of patients with PCI are lower than those of patients with anterior circulation ischaemia (3.93%, 5.3%, 9.7% vs. 7.26%, 9.3%, 13.7%, respectively, *P* < 0.05). The proportion of PCI patients with poor prognosis a year after the onset is also lower than that of patients with anterior circulation ischaemia (6.5% vs. 15.2%, *P* < 0.05) [17]. This study found that different circulatory infarctions are poor predictors of the prognosis of acute cerebral infarction. The prognosis of different circulatory infarctions was different (*P* < 0.05). The prognosis of ACI was worse than that of PCI (OR value, 2.180; 95% CI, 1.286–3.695; *P* value, 0.004 < 0.01); the prognosis of patients with ACI–PCI was better than that of patients with ACI or PCI alone (mean *P* value > 0.05). In terms of prognostic factors, aetiology, clinical manifestations and prognosis, many scholars believe that PCI is different from ACI [3, 18]. Clinicians should treat patients with ACI, PCI or ACI–PCI based on specific causes and prognostic factors rather than the location of the infarction itself [19]. Multivariate analysis of this study showed that different circulatory infarctions have different prognostic factors. This result can serve as a basis for clinicians to estimate the prognosis and perform secondary prevention to shorten the length of hospital stay and thus improve the prognosis.

Given that this study is a single-centre study, the number of samples is limited. Moreover, the vessel segment (M1, M2, etc.) of the occlusion site was not specifically analyzed in this study. It is necessary to further study a larger sample size and analyze the location of vascular occlusion in order to better confirm the results of the current study.

## Conclusions

Diabetes, the Barthel index at admission and previous cerebral infarction are poor prognostic factors of acute cerebral infarction. The prognosis of patients with ACI–PCI does not differ significantly from that of patients with ACI or PCI alone. The prognosis of ACI is worse than that of PCI. Different factors affect the prognosis of different circulatory system infarctions.

## Data Availability

The data that support the findings of this study are available from the corresponding author upon reasonable request.

## Abbreviations

mRS: Modified Rankin Scale
NIHSS: National Institutes of Health Stroke Scale
ROC: The receiver operating characteristic curve.
AUC: the area under the curve
BI: Barthel index
ACI: Anterior circulation infarction
PCI: Posterior circulation infarction
ACI-PCI: Anterior and posterior circulation infarction
TIA: Transient ischemic attack
CHF: Chronic heart failure
AF: Atrial fibrillation
CHD: Coronary heart disease

## References

[1] Lawrence ES, Coshall C, Dundas R, Stewart J, Rudd AG, Howard R (2001). Estimates of the prevalence of acute stroke impairments and disability in a multiethnic population. Stroke.

[2] Zhou M, Wang H, Zhu J, Chen W, Wang L, Liu S (2016). Cause-specific mortality for 240 causes in China during 1990–2013: a systematic subnational analysis for the Global Burden of Disease Study 2013. Lancet.

[3] Di Carlo A, Lamassa M, Baldereschi M, Pracucci G, Consoli D, Wolfe CD (2006). Risk factors and outcome of subtypes of ischemic stroke. Data from a multicenter multinational hospital-based registry. The European Community Stroke Project. J Neurol Sci.

[4] De Marchis GM, Kohler A, Renz N, Arnold M, Mono ML, Jung S (2011). Posterior versus anterior circulation strokes: comparison of clinical, radiological and outcome characteristics. J Neurol Neurosurg Psychiatry.

[5] Wahlgren N, Ahmed N, Eriksson N, Aichner F, Bluhmki E, Dávalos A (2008). Multivariable analysis of outcome predictors and adjustment of main outcome results to baseline data profile in randomized controlled trials: Safe Implementation of Thrombolysis in Stroke-MOnitoring STudy (SITS-MOST). Stroke.

[6] Ntaios G, Papavasileiou V, Faouzi M, Vanacker P, Wintermark M, Michel P (2014). Acute imaging does not improve ASTRAL score’s accuracy despite having a prognostic value. Int J Stroke.

[7] Davignon J, Ganz P (2004). Role of endothelial dysfunction in atherosclerosis. Circulation.

[8] Gruther W, Pieber K, Steiner I, Hein C, Hiesmayr JM, Paternostro-Sluga T (2017). Can early rehabilitation on the general ward after an intensive care unit stay reduce hospital length of stay in survivors of critical illness? A randomized controlled trial. Am J Phys Med Rehabil.

[9] Cipolla MJ, Porter JM, Osol G (1997). High glucose concentrations dilate cerebral arteries and diminish myogenic tone through an endothelial mechanism. Stroke..

[10] Southerland AM, Johnston KC (2012). Considering hyperglycemia and thrombolysis in the Stroke Hyperglycemia Insulin Network Effort (SHINE) trial. Ann N Y Acad Sci.

[11] Li QX, Zhao XJ (2020). Value of the Barthel scale in prognostic prediction for patients with cerebral infarction. BMC Cardiovasc Disord.

[12] Streifler JY, den Hartog AG, Pan S, Pan H, Bulbulia R, Thomas DJ (2016). Ten-year risk of stroke in patients with previous cerebral infarction and the impact of carotid surgery in the Asymptomatic Carotid Surgery Trial. Int J Stroke.

[13] Flossmann E, Rothwell PM (2003). Prognosis of vertebrobasilar transient ischaemic attack and minor stroke. Brain.

[14] van der Hoeven EJ, Dankbaar JW, Algra A, Vos JA, Niesten JM, van Seeters T (2015). Additional diagnostic value of computed tomography perfusion for detection of acute ischemic stroke in the posterior circulation. Stroke.

[15] Zhang DP, Ma QK, Zhang JW, Zhang SL, Lu GF, Yin S (2016). Vertebral artery hypoplasia, posterior circulation infarction and relative hypoperfusion detected by perfusion magnetic resonance imaging semiquantitatively. J Neurol Sci..

[16] Caplan LR, Wityk RJ, Glass TA, Tapia J, Pazdera L, Chang HM (2004). New England medical center posterior circulation registry. Ann Neurol.

[17] Tao WD, Kong FY, Hao ZL, Lin S, Wang DR, Wu B (2010). One-year case fatality and disability after posterior circulation infarction in a Chinese hospital-based stroke study. Cerebrovasc Dis.

[18] Kim JS, Nah HW, Park SM, Kim SK, Cho KH, Lee J (2012). Risk factors and stroke mechanisms in atherosclerotic stroke: intracranial compared with extracranial and anterior compared with posterior circulation disease. Stroke.

[19] Zeng Q, Tao W, Lei C, Dong W, Liu M (2015). Etiology and risk factors of posterior circulation infarction compared with anterior circulation infarction. J Stroke Cerebrovasc Dis.

